# Interventional Treatment of Abdominal Compartment Syndrome during Severe Acute Pancreatitis: Current Status and Historical Perspective

**DOI:** 10.1155/2016/5251806

**Published:** 2015-12-29

**Authors:** Dejan V. Radenkovic, Colin D. Johnson, Natasa Milic, Pavle Gregoric, Nenad Ivancevic, Mihailo Bezmarevic, Dragoljub Bilanovic, Vladimir Cijan, Andrija Antic, Djordje Bajec

**Affiliations:** ^1^Clinic for Digestive Surgery, Clinical Center of Serbia, Faculty of Medicine, University of Belgrade, Belgrade, Serbia; ^2^Department of Surgery, University Hospital Southampton, Southampton, UK; ^3^Institute for Biostatistics and Informatics, Faculty of Medicine, University of Belgrade, Belgrade, Serbia; ^4^Clinic for Emergency Surgery, Emergency Center, Clinical Center of Serbia, Faculty of Medicine, University of Belgrade, Belgrade, Serbia; ^5^Clinic for Surgery, Military Medical Academy, Belgrade, Serbia; ^6^Clinic for Surgery, Clinical Center “Bezanijska Kosa”, Faculty of Medicine, University of Belgrade, Belgrade, Serbia; ^7^Clinic for Surgery, Clinical Center “Zvezdara”, Belgrade, Serbia

## Abstract

Abdominal compartment syndrome (ACS) in patients with severe acute pancreatitis (SAP) is a marker of severe disease. It occurs as combination of inflammation of retroperitoneum, visceral edema, ascites, acute peripancreatic fluid collections, paralytic ileus, and aggressive fluid resuscitation. The frequency of ACS in SAP may be rising due to more aggressive fluid resuscitation, a trend towards conservative treatment, and attempts to use a minimally invasive approach. There remains uncertainty about the most appropriate surgical technique for the treatment of ACS in SAP. Some unresolved questions remain including medical treatment, indications, timing, and interventional techniques. This review will focus on interventional treatment of this serious condition. First line therapy is conservative treatment aiming to decrease IAP and to restore organ dysfunction. If nonoperative measures are not effective, early abdominal decompression is mandatory. Midline laparostomy seems to be method of choice. Since it carries significant morbidity we need randomized studies to establish firm advantages over other described techniques. After ACS resolves efforts should be made to achieve early primary fascia closure. Additional data are necessary to resolve uncertainties regarding ideal timing and indication for operative treatment.

## 1. Introduction

The morbidity and mortality of patients with severe acute pancreatitis (SAP) remain high despite significant improvement in treatment due to the better understanding of the pathophysiology of disease, early aggressive fluid resuscitation, timely surgical intervention, permanent monitoring, and organ supporting intensive care. It is widely accepted that major predictors of unfavorable outcome are development of persistent organ failure and presence of bacterial infection of necrosis [1].

Abdominal compartment syndrome (ACS) in patients with severe acute pancreatitis (SAP) is a sign of severe disease with high risk of adverse outcomes [2–5]. Abdominal compartment syndrome is defined as a state of serious organ dysfunction resulting from sustained increase in intra-abdominal pressure [6]. Very recently published systematic review on this topic showed that the mortality rate in patients who developed ACS during SAP was 49%, while it was 11% in patients without this complication [7]. The number of patients with ACS may have increased as a consequence of a move towards later intervention and minimally invasive rather than open surgery. Several studies demonstrated that development of organ failure in SAP is in correlation with presence of intra-abdominal hypertension (IAH) [3, 4, 8, 9]. Elevated intra-abdominal pressure (IAP) is well known predictor of mortality and serial measurements of IAP are recommended for all patients with severe acute pancreatitis in the intensive care units [6].

Currently there is no agreed surgical or other interventional treatment for ACS during the course of SAP. The World Society of Abdominal Compartment Syndrome (WSACS) has published definitions of IAH and ACS and recommendations for treatment [6], but it is not known if these can be applied to patients with SAP.

During recent years, several studies on ACS in patients with SAP have been published, but data of this problem still remains scarce [3, 10–13]. Some unresolved questions including medical treatment, indications, timing, and interventional techniques remain, and this review will focus on interventional treatment of this serious condition.

## 2. Pathophysiology

There are several reasons which may contribute to development of IAP during SAP. Inflammation of the pancreas is a crucial step, which starts a cascade of events including visceral edema, ascites, acute peripancreatic fluid collections, paralytic ileus, and duodenal obstruction causing gastric dilatations. Aggressive fluid resuscitation, a very important part of initial conservative treatment, is an additional factor leading to rapid fluid accumulation in the abdominal cavity which plays a role in elevation of IAP. Severe intra-abdominal inflammation together with capillary leakage further contributes to development of large quantity of ascites. The abdominal wall may also be edematous with decreased compliance, which in synergy with enlarged intra-abdominal volume leads to an increase of IAP. The presence of large peripancreatic fluid collections and paralytic ileus may also play a significant role in development of IAH.

Intra-abdominal hypertension leads to reduction of chest wall compliance and hypoperfusion of the gastrointestinal tract [14] which contribute significantly to the pathogenesis of organ dysfunction [15, 16]. Elevated IAP may reduce perfusion of abdominal organs, allowing hypoxic injury of the surrounding tissues which could exacerbate systemic inflammatory response. High IAP in patients with severe acute pancreatitis correlates with the degree of organ dysfunction and intensive care stay [9].

An IAP above 20 mmHg is associated with oliguria and significant reduction in cardiac output [17]. IAH appears to exacerbate organ failure, as it is associated with significantly higher APACHE II scores and MODS scores in patients with SAP [4, 5]. De Waele et al. [8] reported a higher incidence of respiratory, circulatory, and renal failure among the patients with IAH. Elevation of the diaphragm due to high IAP may lead to a decrease in lung and chest wall compliance and decline in functional residual capacity and residual volume and may cause respiratory insufficiency. High IAP may cause decrease in renal perfusion pressure, the filtration gradient, and renal blood flow, resulting in renal failure [9]. SAP is characterized by reduced pancreatic perfusion, and it is likely that IAH exacerbates this pancreatic hypoperfusion and consequently increases the extent of pancreatic necrosis. Splanchnic circulation may be decreased due to reduced cardiac output and increased mechanical pressure to splanchnic area [14].

## 3. Medical Treatments to Reduce IAP

Critically ill patients with acute pancreatitis have a considerable risk for developing IAH and routine measuring of IAP is recommended by WSACS guidelines, allowing identification of patients at risk of ACS [6]. If increased IAP is diagnosed, the first option in the management of the patients with IAH during SAP is always nonoperative treatment. This includes management in ICU and neuromuscular blockade with artificial respiration. In a study including 74 patients with SAP, 20 patients developed ACS during first 7 days of admission [3]. Seven patients responded to nonoperative therapy, but the remaining 13 patients had progressive deterioration of organ dysfunction and received interventional decompressive procedure.

Relief of pain and anxiety is an important part of treatment of patients with SAP, but it remains unclear whether this has any influence on IAH [6]. Neuromuscular blockers may reduce IAP by reduction of abdominal musculature tone and an increase in abdominal compliance.

Neuromuscular blockade is the major nonoperative measure in the management of IAH [18]. Since body positioning may alter IAP, the head of the patient's bed should be raised not more than 30 degrees. Nasogastric/colonic decompression via tubes should be established in presence of gastric or colon dilatation. In addition, some reports show a beneficial effect of neostigmine for the treatment of paralytic ileus associated with IAH [19]. Protocol to try to avoid positive cumulative balance is suggested by WSACS guidelines, but it remains uncertain whether use of diuretics and albumin and renal replacement therapy improve outcomes [6].

## 4. Interventional Treatment

When medical treatment fails to relieve IAH and ACS has developed, interventional measures seem to be necessary. First line therapy for those patients should be insertion of a percutaneous drainage catheter (PCD) under radiological guidance which can relieve ACS [20–22]. In a randomized study to compare the effects of indwelling catheter and conservative measures in the treatment of ACS in fulminant acute pancreatitis, Sun et al. [20] found that intra-abdominal pressure was positively correlated with drainage volume, duration of hospitalization time, and APACHE II score. Outcomes (relief of abdominal pain and hospitalization time) were significantly better in patients with abdominal catheter than in those treated conservatively. The observed reduction in mortality rate from 20.7% to 10% was not statistically significant.

In the previously mentioned systematic review including 103 patients with ACS, the authors found that PCD was performed as first line therapy in only 13% of patients [7]. In addition, surgical decompression was performed in 73% of patients in whom PCD was firstly inserted. Since these data come from observational retrospective and prospective studies with moderate to low methodological quality, it still remains uncertain why PCD was not performed in a bigger portion of patients. In addition, the time frames between diagnoses of SAP to ACS, as well as values of any organ scoring system in the moment of intervention, were not reported.

### 4.1. Indications and Timing of Interventional Treatment

Still there is no clear consensus on the optimal surgical management in patients with ACS during SAP. Data defining the optimal surgical procedure, indication, and timing of surgery in these patients are lacking. WSACS clinical practice guidelines “recommend decompresive [*sic*] laprotomy [*sic*] in cases of overt ACS compared to strategies that do not to use decompressive laparotomy…” [6]. There is no explanation on which approach should be used for laparotomy and what exactly means overt ACS (level of IAP, degree of systemic complications, and so forth).

Timing of abdominal decompression remains uncertain in the treatment of these patients. However the Finnish group published their experience regarding early and late decompression [10]. Results clearly showed that early decompression (first 4 days) was associated with significantly less deaths, compared with late decompression (after 4 days). Davis et al. [12] reported 25% mortality in surgically decompressed patients with ACS. Mean time from diagnosis to surgical intervention in their study was 3.1 h. It seems that early surgical decompression in patients with ACS during SAP may be associated with less mortality rate.

According to current literature, several surgical approaches have been described in order to improve outcome of patients who develop ACS during SAP. Some procedures have been reported that could be valuable and applicable in these patients. Decompressive laparotomy with subsequent laparostomy for the treatment of ACS has been used most frequently [2, 7, 10–13, 16]. The most commonly performed approach for full-thickness laparostomy is midline incision [23]. However, some authors suggested that bilateral subcostal incision has potential advantage such as easier and safer abdominal wall reconstruction [10, 24]. During both procedures, the abdominal wall is mainly reconstructed using some technique for temporary abdominal closure. For this purpose several types of abdominal zippers, plastic silo bag (Bogota bag), or vacuum assisted closed system were used. Temporary abdominal closure carries several advantages in comparison to leave abdomen open which comprises minimizing fluid and protein losses from the wound, prevention retraction of fascia edges, maintaining the abdominal domain, and avoiding the scenario of “hostile abdomen” [25].

When ACS is resolved using decompressive laparotomy, the most important goal is to obtain primary fascia closure. WSACS guidelines recommended protocolized efforts to achieve an early or at least same hospital stay fascial reconstruction [6]. However, this guideline did not make recommendation regarding use of an acute component separation technique to allow early abdominal fascial closure [6]. In addition, it did not suggest routine use of bioprosthetic mesh to facilitate early abdominal wall reconstruction. It seems that, without accumulative data regarding several techniques described, gradual fascial closure is an acceptable approach for early abdominal closure. When patient's condition does not allow primary fascia closure, skin coverage could be a good alternative. For this purpose, split-thickness skin graft or microvascular flaps for large defects could be used [25].

Several investigators also suggested skin incisions to perform a subcutaneous fasciotomy with the peritoneum left intact. Two techniques have been described: subcutaneous linea alba fasciotomy [26] and fasciotomy of the anterior rectus abdominis sheath [27]. The rationale for introducing these alternative methods was to avoid complications of open abdomen management. These approaches were reported in very limited group of patients and were associated with significant decrease in IAP levels. Risk of development of recurrent ACS is high and it should be monitored closely. Lower incidence of infection of pancreatic necrotic tissue and fistulas and no need for additional treatment connected to open abdomen are potential benefits, although a high proportion of these patients require additional surgery for ventral hernia repair. These approaches deserve attention, but we need more data to support implementation in daily practice.

## 5. Surgical Treatment

According to WSACS guidelines, in patients with persisting ACS despite PCD procedure performed, decompressive laparotomy is recommended [6].

First steps in surgical management for patients with ACS during SAP have been reported. Gecelter et al. [2] published 13 years ago their experience with 3 patients suffering from ACS during SAP who were surgically decompressed with mortality rate of 67%. In conclusion, the authors pointed out that this issue “has been ignored by current surgical literature.” In the title of their paper they put question mark: “Abdominal compartment syndrome in severe acute pancreatitis: an indication for a decompressing laparotomy?”. In 2005 Wong and Summerhays [28] presented case report and discussed the diagnosis and treatment of ACS as a new indication for operative intervention in SAP. In the same year, De Waele et al. [8] analyzed 44 patients with SAP of whom 4 received abdominal decompression due to ACS. In one patient necrosectomy was done during the decompressive laparotomy. Mortality rate in decompressed group was 75% and the authors concluded that it is not clear if surgical decompression in these patients is advantageous.

Among the 13 patients with ACS who did not respond to medical and PCD therapy, in 8 decompressive laparotomy was performed [3]. After laparotomy abdomen was temporarily reconstructed with silastic covering obtained from a sterilized inner surface of an intravenous bag sewn to the fascia, scheduled closure of abdominal wall was attempted as soon as acute episode resolved and edema and inflammatory collections reduced significantly. The authors did not report complications directly related to decompressive laparotomy. In this study, IAP decreased from 37 mmHg before decompression to 18 mmHg after procedure. In addition, the high peak airway pressure improved significantly, as well as other general physiologic parameters including serum pH, base excess, lactate, and mean arterial pressure.

Boone et al. [13] investigated the outcomes of 12 patients with SAP who underwent decompressive laparotomy for treatment of ACS during 9-year period. The laparotomy was performed within 4.5 days after disease onset and 4 patients were operated on in intensive care unit due to cardiopulmonary instability. Statistically significant improvements were noticed in several physiologic parameters. However, despite initial improvement almost in all patients, mortality rate of 50% was recorded. Authors concluded that patients with this highly lethal complication may have benefited from early surgical decompression.

There have been several case reports in the literature with high early mortality rate, ranging from 17 to 75% [3–6, 8, 9, 23]. A high proportion of patients in these reports, during surgical decompression, received retroperitoneal debridement and early mortality was mainly associated with uncontrolled retroperitoneal bleeding [8]. There is no evidence in the literature for early debridement of necrotic area during surgical decompression. Very limited experience supported the strategy of decompressive laparotomy in patients with ACS during SAP but without premature exploration of pancreatic region and retroperitoneum [23]. All these data have not provided enough clear evidence to support a treatment algorithm for ACS in patients with SAP, although two approaches deserve more attention than the others. These are decompressive laparotomy with temporary abdominal closure and percutaneous puncture with placement of abdominal catheter. Both of these procedures raise several unresolved issues for decompressive laparotomy: (a) the relation to potential necrosectomy, (b) difficulties in management of semiopen abdomen, (c) increased risk of enteric fistulas, (d) potentially higher number of patients with infected pancreatic necrosis than expected, and (e) incidence of postoperative hernias. The main unanswered question for percutaneous puncture with placement of abdominal catheter is whether it is possible to achieve sufficient decompression and relief of ACS using this procedure. In 2010, Belgrade group initiated multicenter, randomized, controlled study “Decompressive Laparotomy with Temporary Abdominal Closure versus Percutaneous Puncture with Placement of Abdominal Catheter in Patients with Abdominal Compartment Syndrome during Acute Pancreatitis” [29]. The rationale for this study was that during that time decompressive laparotomy for ACS associated with SAP has not been studied in large patients group. So far, 79 patients have been randomized and we expected that we will finish with patients recruitment until the end of this year.

## 6. Other Surgical Approaches

In a retrospective study Helsinki group showed results of surgical decompression in 26 consecutive patients with ACS during SAP [10]. In the biggest series published so far, different surgical decompressive methods were performed including full-thickness midline laparostomy, full-thickness transverse subcostal bilateral laparostomy, and subcutaneous linea alba fasciotomy. Bogota bag and vacuum assisted closure (VAC) were used for temporary abdominal closure. IAP decreased by 16 mmHg in patients who received full-thickness laparostomy and by 12 mmHg in those with line alba fasciotomy. Mean SOFA score before interventions was 12 but did not improve significantly during the first 5 postoperative days. The median number of reoperation was 4 and fistulas developed in 4 patients. All of these patients received either necrosectomy or bowel resection. The mortality rate in this study was 46%. More importantly, mortality rate in the group of patients who received decompressive surgery after day 5 after disease onset was 100%, in comparison with 18% in those who were operated on within first 4 days of disease. Results of this study clearly showed that early surgical decompression is more effective than late surgical decompression and that in patients with early MODS and ACS during SAP surgical intervention is indicated.

Subcutaneous linea alba fasciotomy (SLAF) as a minimally invasive treatment method for ACS was analyzed in a retrospective study including 10 patients with SAP [26]. The decrease of IAP after procedure was 10 mmHg. The value of SOFA score did not decrease in nonresponder patients, while in those with successful SLAF it decreased by five or more points. Four patients required a completion laparostomy within 24 hours. The mortality rate of 40% was noticed in this study. There were no complications related to SLAF. According to results it was concluded that SLAF is a safe decompressive technique and that in nonresponders a completion laparostomy is required.

The feasibility and effectiveness of subcutaneous fasciotomy of the anterior rectus abdominis sheath were assessed in 3 patients with ACS during SAP [27]. Surgical intervention was performed within 8 hours after admission to ICU and the diagnosis of ACS was confirmed. Decrease of IAP from 25 to 16 mmHg (mean values) was noticed, while MODS and APACHE II score were very slightly lower after intervention (from 6 to 5.3 points and from 12.3 to 10.6 points, resp.). Despite the effective control of IAH in all patients, systemic complications persisted, and two patients died. This report showed that subcutaneous fasciotomy of the anterior rectus abdominis sheath could decrease IAP, but effectiveness should be checked in carefully prepared prospective studies.

Deng et al. [11] reported experience of 8 patients with ACS during SAP which was surgically decompressed. During surgical intervention a catheter for antibiotic, octreotide, and protease inhibitor application was inserted into peripancreatic artery. APACHE II score decreased from 18 to 5.4 points, while IAP decreased from 29 mmHg to 7.7 mmHg. Mortality rate was amazingly low, 12.5%.

## 7. Complicating Factors for Surgical Decompression

A study dealing with the impact of obesity and decompressive laparotomy on mortality in SAP patients was published very recently [12]. Decompressive laparotomy was performed in 16 patients and the abdominal wall was reconstructed either with Bogota bag (11 patients) or with VAC system (5 patients). The mean BMI in this group was 30.3 kg/m^2^, while 63% of patients were obese with BMI higher than 30 kg/m^2^. Time interval between diagnosis of ACS and decompressive laparotomy was 3.1 h. Substantial morbidity was noticed including development of fistulas in 43% of patients, 18% of patients required the use of split-thickness skin graft for the closure of their incision, and 50% developed incisional hernias that required delayed repair. This approach was associated with mortality rate of 25%. Interestingly, according to results of this study, obesity was not predictive for development of ACS or mortality, nor was the presence of ACS a predictor of fatal outcome. Similar death rates were seen in patients who required decompressive laparotomy and those treated without surgery, indicating that in selected patients this procedure might be promising.

## 8. Conclusions

Abdominal compartment syndrome is a well-recognized clinical entity that significantly influences outcome of patients with SAP. It occurs as combination of inflammation of retroperitoneum, visceral edema, ascites, acute peripancreatic fluid collections, paralytic ileus, and aggressive fluid resuscitation. First line therapy for this life-threatening complication is conservative treatment aiming to decrease IAP and to restore organ dysfunction. If nonoperative measures are not effective, early abdominal decompression is mandatory. Midline laparostomy seems to be method of choice. Since it carries significant morbidity we need randomized studies to establish firm advantages over other described techniques. After ACS resolves, efforts should be made to achieve early primary fascia closure. Additional data are necessary to resolve uncertainties regarding ideal timing and indication for operative treatment.
